# Current Data on *Rickettsia felis* Occurrence in Vectors, Human and Animal Hosts in Europe: A Scoping Review

**DOI:** 10.3390/microorganisms10122491

**Published:** 2022-12-16

**Authors:** Constantina N. Tsokana, Ioanna Kapna, George Valiakos

**Affiliations:** Faculty of Veterinary Science, University of Thessaly, 43100 Karditsa, Greece

**Keywords:** arthropods, hosts, One Health, prevalence, rickettsiosis, zoonosis

## Abstract

*Rickettsia felis* is an emerging pathogen with increasing reports of human cases and detection in arthropod and animal host species worldwide. In this scoping review we record the newest data reported for *R. felis* in Europe: the vector and host species found to be infected, and the geographical distribution and prevalence of *R. felis* infection in vectors and hosts. A total of 15 European countries reported the occurrence of *R. felis* in hosts and vectors during 2017–2022. The vectors found to be infected by *R. felis* were flea, tick and mite species; *Ctenocephalides felis* and *Ixodes ricinus* were the dominant ones. The hosts found to be infected and/or exposed to *R. felis* were humans, cats and small mammals. Physicians should be aware of the epidemiology and include illness caused by *R. felis* in the differential diagnosis of febrile disease. Veterinarians should keep training pet owners on the need for effective year-round arthropod control on their pets, especially for fleas.

## 1. Introduction

*Rickettsia felis* in an obligate intracellular Gram negative bacterium and the causative agent of flea-borne spotted fever (FBSF) [1]. Although being originally characterized as a typhus-like *Rickettsia* belonging in the typhus group (TG) [2], its classification is still debated—in the spotted fever group (SFG) by some and to the more recent transitional group (TRG) by others [3,4].

*Rickettsia felis* requires a vertebrate and invertebrate host to survive and reproduce. The cat flea (*Ctenocephalides felis*) is considered as the primary vector and the reservoir host of this pathogen [2,5]. *Rickettsia felis* has been also identified in various flea species and there is a growing evidence of detection in other arthropods: ticks, mites, lice and mosquitoes. Similarly, the host range of *R. felis* is increasing; reports on infected humans, domestic and wild animals are coming from all over the world. However, the competency of the different arthropods and hosts as vectors and reservoirs, respectively, is yet to be demonstrated [5].

*Rickettsia felis* follows the distribution of its vector; it occurs on all continents except Antarctica [6]. The first human case was reported in Texas in 1994 [7] and the first autochthonous human case was reported in Europe in 2002 [8], suggesting that this pathogen was not restricted to USA and it had the potential for global distribution. The lack of specific diagnostics and the similarity of FBSF with the disease caused by *R. typhi* [Flea-borne (murine) typhus] or with other vector-borne diseases, potentially leads to the under-diagnosis of the disease caused by *R. felis.* Thus, the true number of *R. felis* cases may be under-estimated. Under-reporting may also be enhanced by the self-limiting nature of the disease [5].

Although originally considered a sporadic disease, febrile illness has recently been regularly associated with *R. felis* in sub-Saharan Africa; the monthly incidence of *R. felis* infection in humans was found to reach approximately 17% during spring [9,10]. The recent identification of *R. felis* in the literature, and the increasing number of human cases from different regions in parallel to the fast-growing reports of the worldwide detection of *R. felis* in different arthropod and host species, justify its designation as an emerging pathogen [5,11,12].

The majority of the research on *R. felis* is undertaken in America, Africa and Asia. However, *R. felis* is an emerging pathogen of public health importance in Europe and both physicians and veterinarians should be aware of its epidemiology and distribution. This scoping review summarizes the knowledge obtained on the occurrence of *R. felis* in European countries as recorded by published studies during the last five years. Our aim is to identify and analyze the published data and to determine the extent of the research on this topic in Europe, and the prevalence of *R. felis* infection in different areas and vector and host populations. In this study, we followed the Preferred Reporting Items for Systematic Reviews (PRISMA) guidelines as an approach to collect relevant data from electronic databases (PubMed, Google Scholar and Scopus) [13]. The objectives of this study were to record the newest data reported in Europe from 2017–2022 for *R. felis* in terms of: (1) the vector and host species infected by *R. felis*; (2) the geographical distribution of *R. felis* infected vectors and hosts; (3) the prevalence of *R. felis* infection and/or exposure in vectors and hosts.

## 2. Vectors and Hosts of *R. felis* in Europe

### 2.1. Vectors

**Figure 1:**
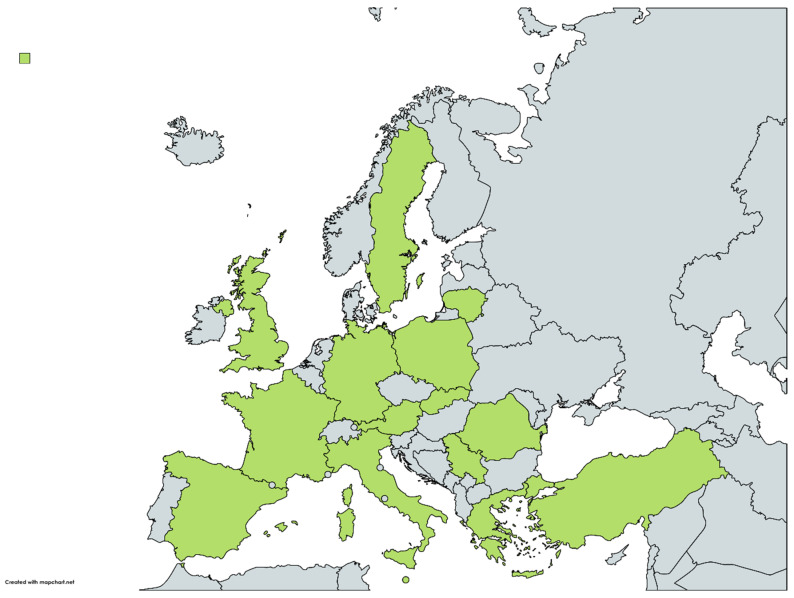
Map showing the European countries (in green) that reported the occurrence of *R. felis* during 2017–2022 in hosts and vectors (https://www.mapchart.net/europe.html, accessed on 12 November 2022).

During 2017–2022, a total of 11 European countries reported the occurrence of *R. felis* in several vector species (Figure 1). The vectors found to be infected included flea, tick and mite species; the dominant flea and tick species were *C. felis* and *I. ricinus*, respectively. The baseline characteristics of the studies on vectors which were included in this review are tabulated in Table 1 and are presented below in detail.

#### 2.1.1. Fleas

##### Austria

In the only relevant study derived from Austria, the researchers investigated the occurrence of *R. felis* in 105 *C. felis* samples collected from 39 free-roaming or stray cats in the eastern part of the country. The small number of positive samples (1/105) did not allow for the determination of the prevalence of *R. felis* in cat fleas [14].

##### France

The 2021 study by Zurita et al. reported negative molecular results for the detection of *R. felis* in 105 flea samples belonging to three species (*Nosopsyllus fasciatus*, *Stenoponia tripectinata* and *Leptopsylla taschenbergi)* collected from rodents of the *Rattus* spp., *Mus* spp. and *Apodermus* spp. in France during 2011–2018 [15].

##### Greece

Two studies were carried out in Greece on the occurrence of *R. felis* in fleas. In the study of Chochlakis et al., the researchers investigated the occurrence of *R. felis* in different vectors around the residences of patients presented to Greek hospitals (described in Section 2.2.1) during 2010–2013. The flea species included in this study were *Xenopsylla cheopis* from rats (*Rattus norvegicus*) and *C. felis* from cats (*n* = 23) and dogs (*n* = 11). *R. felis* was identified only in three *C. felis* samples collected from cats (3/23, 13%) [16].

Later, Dougas et al. reported the detection of *R. felis* in fleas from owned cats and dogs during 2016–2017 in the region of Attica, Greece, and compared the efficiency of various molecular techniques for *R. felis* detection. The researchers included 100 female flea pools in the study, collected from 67 cats and 33 dogs. *C. felis* was the dominant flea species among those identified (*C. felis*, *C. canis* and *Pulex irritans*). *Rickettsia felis* was detected in 14 out of the 100 flea pools [17].

##### Lithuania

The first report of *R. felis* in Lithuania came from the 2018 study by Radzijevskaja et al. The authors were also the first to report *R. felis* in *Ctenophthalmus agyrtes* and *Hystrichopsylla talpae* fleas. A total of 115 fleas belonging to eight species (*Ct. agyrtes*, *Ct. assimilis*, *H. talpae*, *H. orientalis*, *Megabothris turbidus*, *M. walkeri*, *Palaeopsylla soricis* and *N. fasciatus*) were collected from 238 rodents during 2013–2014. Almost 44% of the fleas originating from five rodent species (*Apodemus flavicollis*, *Myodes glareolus*, *Micromys minutus*, *Microtus oeconomus* and *M. agrestis*) were found to be infected by *Rickettsia* spp. Four *Rickettsia* spp. were identified in fleas. Among them, *R. felis* was detected in *Ct. agyrtes* and *H. talpae* fleas from *A. flavicollis*. The *R. felis* prevalence in flea rodent species was not defined [18].

##### Malta

Two studies conducted in the island of Malta demonstrated the high prevalence of *R. felis* in cat fleas.

The study, carried out in 2017, reported for the first time the detection of *R. felis* in fleas collected from cats in Malta. A total of 38 fleas from 11 cats were molecularly examined and *R. felis* DNA was identified in 39.47% of the examined fleas (15/38), suggesting a relatively high epidemiological risk for human infection in this region [19].

Similarly, a high prevalence of *R. felis* infection in fleas collected from cats was reported by Mifsud et al. in the southern part of the island of Malta. In 2017, the researchers collected a total of 207 fleas from 56 cats living in a shelter. *Ctenocephalides felis* was the dominant species while *C. canis* was also identified in one cat. Among other detected pathogens with zoonotic potential, *R. felis* was the most prevalent; the pathogen DNA was detected in 96.42% (54/56) of pooled flea samples [20].

##### Slovakia

The presence of *R. felis* in fleas collected from small mammals in Slovakia was documented in two studies that included sampling from different habitat types with variable anthropogenic impact.

The 2020 study by Heglasová et al. was carried out in Eastern Slovakia during 2014–2016 and included 279 fleas. The flea samples were collected from 250 small mammals belonging to eight species (*A. agrarius*, *A. flavicollis*, *A. uralensis*, *M. glareolus*, *M. arvalis*, *M. subterraneus*, *Crocidura leucodon*, and *Sorex minutus*); 46% of them were found to be infested by fleas. Urban, suburban and rural habitats were targeted for sampling, with the latter showing the greatest flea abundance and diversity. Generalists (*Amalareus penicilliger*, *Ct. agyrtes* and *M. turbidus*) and flea species with a narrower host range (*Ct. solutus*, *Ct. uncinatus*,) were found to be infected by *Rickettsia* spp. at a low rate (7/279, 2.5%). *Rickettsia felis* was detected in only one *C. solutus* female flea collected from a *Rickettsia*-negative *A. agrarius* inhabiting an urban area [21].

Earlier data on *R. felis* detection in fleas from small mammals comes from a study conducted in Slovakia during 2012–2014 with samples originating from three habitats: suburban, natural and rural. A total of 665 fleas belonging to 12 species were collected from 640 small mammals from 6 species (*A. flavicollis*, *A. sylvaticus*, *M. glareolus*, *M. arvalis*, *M. subterraneus*, and *M. minutus*). In this case, the suburban habitat presented the highest prevalence of flea infestation but the total prevalence of infestation was 45.47%. The overall *Rickettsia* spp. infection rate was 19.1% (127/665) and extremely variable between the different habitats, ranging from 0.86% in the natural to almost 77.27% in the rural habitat. Among other *Rickettsia* spp. identified, two specimens—one *N. fasciatus* and one *C. assimilis*—collected from the rural habitat, harbored *R. felis* [22].

##### Spain

Two studies conducted in Spain reported the occurrence of *R. felis* in fleas. The 2020 study by Abreu-Yanes et al. was the first to describe a high prevalence of *R. felis* in *C. felis* in Tenerife, the Canary Islands, Spain. A total of 128 *C. felis* samples were collected from stray and sheltered cats (*n* = 101) and dogs (*n* = 27) during 2019–2020. *Rickettsia* spp. DNA was molecularly detected in 37.5% (48/128) of fleas. Out of the 48 positive samples, 38 were successfully sequenced and identified as *R. felis*. Co-infections of *R. felis* with *Bartonella henselae* and *B. clarridgeiae* were also demonstrated in this study [23].

Zurita et al. reported for the first time the detection of *R. felis* in *Ct. b. boisseauorum*, with a 1.6% prevalence of infection. The respective infection prevalence was 28.3% in *C. felis* and 33.3% in *Archaeopsylla erinacei.* The study was carried out in Asturias (north of Spain) and Andalusia (south of Spain) during 2011–2018 and included a total of 214 fleas belonging to five species (*C. felis*, *P. irritans*, *C. apertus allani*, *A. erinacei* and *Ct. b. boisseauorum*) from dogs (*n* = 6), horse stables (*n* = 1), hedgehogs (*n* = 3) and *Arvicola terrestris* rodents (*n* = 29) [15].

##### UK

Data on a 5.7% *R. felis* prevalence in pooled flea samples (*n* = 470) from cats (*n* = 227) and dogs (*n* = 94) come from a single study conducted in the UK in 2018. Among the five flea species identified in this dog and cat population (*C. felis*, *C. canis*, *A. erinacei*, *Spilopsyllus cuniculi*, and *Ceratophyllus* spp.), *C. felis* was the most prominent species and *R. felis* DNA was detected in *C. felis* (*n* = 26) and in one *C. canis*, suggesting that other flea and animal species may also act as vectors of this pathogen and potential reservoirs, respectively [24].

#### 2.1.2. Ticks

##### France

Two studies coming from France investigated the occurrence of *R. felis* in ticks. In the 2019 study of Lejal et al., the research team collected *Ixodes ricinus* ticks in a peri-urban forest for three consecutive years (2014–2017), comprising a total of 998 nymphs. Among the 31 important tick-borne pathogens (TBP) included in the study, *R. felis* was unexpectedly identified in one *I. ricinus* nymph (1/998, 0.1%) while 15.9% of the tested samples were positive for at least one tested pathogen. The authors suggested that sporadic tick samplings are not sufficient to determine TBP prevalence as seasonal and annual fluctuations exist and a unique sampling would certainly not facilitate the detection of *R. felis* [25].

In a previous study, Lejal et al. showed that *I. ricinus* ticks collected from a forest in southern Paris, France, in 2017 were infected with *R. felis* [26]. Interestingly, *R. felis* was detected only in the salivary glands in male and female ticks reaching an infection rate of 7% in this organ. The authors suggested that the exclusive pathogen location in the salivary glands may probably be associated with the speed of transmission after the tick bite. Probably, *R. felis* does not remain in the mid-gut and rapidly migrates to the salivary glands, being present for the next blood-feeding [26].

##### Germany

Negative results for *R. felis* were reported in a single study from Germany conducted during 2012–2014. The researchers molecularly examined 474 ticks belonging to three species (*I. ricinus*, *Dermacentor reticulatus*, and *I. trianguliceps*) that have been removed from 673 small mammals belonging to eight species (*A. agrarius*, *A. flavicollis*, *M. arvalis*, *M. agrestis*, *Mustela nivalis*, *M. glareolus*, *S. araneus*, and *Talpa europaea*). Although 24.8% of all examined questing ticks were positive for *Rickettsia* spp, *R. felis* DNA was not detected [27].

##### Greece

Chochlakis et al. reported negative molecular results for *R. felis* detection in different tick species from dogs, sheep and goats (*Rhipicephalus sanguineus* s.l. from dogs, *Rh. turanicus*, *Rh. bursa*, *Hyalomma excavatum*, *Haemaphysalis sulcata* and *Hae. punctate* from sheep and goats) [16].

##### Italy

Two studies reporting the detection of *R. felis* in tick species of the genus *Ixodes* spp. and *Rhipicephalus* spp. come from southern and central Italy. In 2018, Raele et al. chose the National Park of Gargano, in the Apulia region, Italy, to assess the circulation of SFG rickettsiae in ticks, due to its proximity to both wild and domestic animals, mainly ovine; its location within an endemic area for rickettsiosis; and its high level of biodiversity of vertebrate and invertebrate species. In 2013, the research team collected 158 ticks (110 manually removed from dead animals and 48 by the dragging method) and examined them in 34 pools by species and host. Out of the six tick species identified (*I. ricinus*, *I. acuminatus*, *Rh. sanguineus*, *Rh. bursa*, *Rh. turanicus*, and *D. marginatus*), *R. felis* DNA was detected only in one pool (1/34, 2.9%) consisting of five adult *Rh. turanicus* ticks that had been collected from sheep. The low number of positive pools did not allow for the determination of the infection rate but this was the first evidence of *R. felis* detection in *Rh. turanicus,* suggesting the increasing dispersal of this pathogen in very heterogeneous group of vectors [28].

Pascucci et al. reported the detection of *R. felis* in *I. hexagonus* collected from one hedgehog (*Erinaceus europaeus*) and one red fox (*Vulpes vulpes*) from the Abruzzi and Molise regions, Italy, in the context of a passive survey on ticks during 2014–2016. The researchers collected a total of 605 adult ticks belonging to seven tick species (*I. ricinus*, *D. marginatus*, *Rh. sanguineus*, *I. hexagonus*, *Rh. turanicus*, *Hy. marginatum and Hae. punctate)* and originating from 15 different host species (domestic and wild animals and humans). Eight *Rickettsia* species were identified (*R. slovaca*, *R. monacensis*, *R. massiliae*, *R. conorii*, *R. aeschlimannii*, *R. helvetica*, *R. raoultii*, *and R. felis*) in the 178 tick pools examined. Although not being able to determine the infection rate in the case of *R. felis* due to the small positive sample size, the detection of this pathogen in *I. hexagonus* was an original finding and emphasized the need for further investigation [29].

##### Lithuania

The 2018 study by Radzijevskaja et al. reported negative results for *R. felis* detection in *I. ricinus* ticks (*n* = 596) from 238 rodents during 2013–2014. In total, 26.5% of the *I. ricinus* ticks, which originated from five rodent species (*A. flavicollis*, *M. glareolus*, *M. minutus*, *M. oeconomus and M. arvalis*), tested positive—two *Rickettsia* spp. other than *R. felis* were identified [18].

##### Romania

A single study conducted in Romania provided the first evidence of *R. felis* occurrence in the country. The researchers examined questing and engorged ticks from rodents, birds and hedgehogs. *Rickettsia felis* was not detected in questing ticks; *R. helvetica* and *R. monacens* were the dominant species in ticks from both urban and peri-urban areas. Among the different tick species identified, namely *I. ricinus* (*n* = 164), *I. hexagonus* (*n* = 36), *Hae. punctata* (*n* = 16) and *Hae. concinna* (*n* = 6), *R. felis* DNA was detected in only one engorged *I. ricinus* nymph (1/222). Additionally, this study showed a great diversity and prevalence of TBPs in engorged ticks collected from urban sites and a high frequency of co-infections in both questing and engorged ticks [30].

##### Serbia

Two studies reported the circulation of *R. felis* in Serbia in both ticks and humans (see also Section 2.2.1).

In a 2021 study, Banović et al. provided the first molecular evidence of *R. felis* infection in one *I. ricinus* tick out of 31 ticks (3%, 30 *I. ricinus* and one *Rh. sanguineus* s.l.) collected from human patients in 2019. Importantly, both the tick and the patient tested positive for *R. felis.* Although further studies are needed to support its competency, this finding is suggestive of the potential role of *I. ricinus* as a vector for *R. felis* in humans [31].

In 2020, Banović et al. molecularly examined 93 ticks attached to human patients for several TBPs. The tick species identified were *I. ricinus*, *Rh. sanguineus* s.l., *D. reticulatus*, and *Hae. punctate*; almost 67.74% of them were positive for at least one of the tested pathogens. *R. felis* was detected in 4.3% (4/93) of the examined ticks [32].

##### Spain

The first evidence of *R. felis* in *I. ricinus* ticks from Spain comes from a single study conducted during 2015–2017 in the north-western part of the country. The researchers collected a total of 1093 questing ticks belonging to four species: *I. ricinus* (*n* = 1056), *D. marginatus* (*n* = 19), *D. reticulatus* (*n* = 17) and one *I. acuminatus*. *Rickettsia felis* was among the five different *Rickettsia* spp. identified. This study showed that *Rickettsia spp.* are very prevalent in *I. ricinus*, *D. marginatus* and *D. reticulatus* collected from vegetation in north-western Spain. However, the prevalence of *R. felis* was low (0.46%, 1/219) and *I. ricinus* was the only tick species found to be infected by this pathogen [33].

#### 2.1.3. Mites

##### Lithuania

The 2018 study by Radzijevskaja et al. reported for the first time *R. felis* in the mite species *Laelaps agilis* and *Hyperlaelaps microti* removed from *M. minutus*. A total of 550 mites belonging to five different species (*L. agilis*, *H. microti*, *Haemogamassus nidi*, *Eulaelaps stabularis* and *Myonyssus gigas*) were collected from 238 rodents during 2013–2014. In total, 11.0% of single mite specimens and 34.3% of pools (*L. agilis*) originating from four rodent species (*A. flavicollis*, *M. glareolus*, *M. minutus*, and *M. oeconomus*) tested positive for *Rickettsia* spp., all of them being female mites. Three *Rickettsia* spp. were identified in *Laelapidae* mites. The prevalence of *R. felis* in mite rodent species was not defined [18].

**Table 1:** The reported occurrence of *R. felis* in different vectors in Europe (2017–2022).

Countries	Study Period	Vectors	Prevalence in Vector	Vector Hosts	Reference
Austria	2016	*C. felis*	Not defined (1/105)	Cats	[14]
France	2014–2017	*I. ricinus*	0.1% (1/998)	Environment	[25]
France	2017	*I. ricinus*	7% **	Environment	[26]
Greece	2013	*C. felis*	13% (3/23)	Cats	[16]
Greece	2016–2017	*C. felis*, *C. canis*, *P. irritans*	14% (14/100) *	Dogs and Cats	[17]
Italy	2013	*Rh. turanicus*	2.9% (1/34) *	Sheep	[28]
Italy	2014–2016	*I. hexagonus*	Not defined	Hedgehog and fox	[29]
Lithuania	2013–2014	*H. microti*, *L. agilis*, *Ct. agyrtes*, *H. talpae*	Not defined	Rodents	[18]
Malta	2017	*C. felis*	39.47% (15/38)	Cats	[19]
Malta	2017	*C. felis*	96.42% (54/56) *	Cats	[20]
Romania	2018	*I. ricinus*	Not defined (1/222)	Rodents, birds, hedgehogs	[30]
Serbia	2019	*I. ricinus*	3% (1/31)	Humans	[31]
Serbia	2020	Ticks	4.3%	Humans	[32]
Slovakia	2012–2014	*N. fasciatus*, *Ct. assimilis*	Not defined	Rodents	[22]
Slovakia	2014–2016	*Ct. solutus*	Not defined	Small mammals (*A. agrarius*)	[21]
Spain	2011–2018	*C. felis*	28.3% (15/53)	Dogs	[15]
		*A. erinacei*	33.3% (6/18)	Hedgehogs	
		*Ct. b. boisseauorum*	1.6% (1/60)	Rodents (*A. terrestris*)	
Spain	2015–2017	*I. ricinus*	0.46% (1/219)	Environment	[33]
Spain	2019–2020	*C. felis*	29.6% (38/128)	Dogs and Cats	[23]
UK	2018	*C. felis*, *C. canis*	5.7% (27/470) *	Dogs and Cats	[24]

* pooled samples. ** refers to tissue samples.

### 2.2. Hosts

During 2017–2022, a total of nine European countries reported the occurrence of *R. felis* in different hosts (Figure 1). The hosts found to be infected by *R. felis* by molecular methods or exposed to *R. felis* by serology were humans, cats and small mammals. The baseline characteristics of the studies on hosts which were included in this review are tabulated in Table 2 and are presented below in detail.

#### 2.2.1. Humans

##### Germany

In a 2017 study, Wölfel et al. showed that exposure to *Rickettsia* spp. is highly prevalent among forestry workers, a population with an increased risk for tick borne diseases, in the federal state of Brandenburg, in eastern Germany. Specific IgG antibodies against *R. felis* were detected in 2.7% of the participants. The study was carried out in 2008 and included 559 serum samples from men (*n* = 495) and women (*n* = 64). The serum samples were examined using IFA and a microimmunofluorescence (MIF) assay against the five most common rickettsiae in Germany: *R. helvetica*, *R. raoultii*, *R. slovaca*, “*R. monacensis*” and *R. felis*. The forestry workers showed an average of 27.5% seroprevalence against *Rickettsia* spp. that varied significantly between the investigated districts from 11% up to 55% [34].

##### Greece

Chochlakis et al. reported eight human cases of potential exposure to *R. felis* through serology from 2010 to 2013 in Greece. The patients lived close to regions endemic for *R. typhi* (Evoia and Chania, Crete) or regions where SFGR has been described previously (Sitia, eastern Crete). They presented to hospitals with various clinical signs, including fever, and most of them gradually developed a rash. Although the blood samples and eschars tested negative using molecular methods, the IgM titers in IFA ranged from 200 to 400, the IgG titers from 0 to 240, and the convalescent sera showed either seroconversion or a decrease in antibodies 18–25 days later [16].

##### Serbia

During the investigation of the exposure of human patients infested with ticks to TBPs in 2019 in Serbia, Banović et al. reported the detection of *R. felis* DNA in one human blood sample (3%, 1/30). Interestingly, as mentioned previously (see also Section 2.1.2), this *I. ricinus* tick infesting the patient also tested positive for *R. felis* by PCR. The patient was a 71-year-old female, presenting with an adult female tick attached and the approximate feeding time was less than 24 h. The patient was diagnosed with atypical erythema migrans and reported itching at the site of the tick bite, which persisted even after eight days of treatment with doxycycline [31].

Following the previous study, Banović et al. recruited 85 patients with at least one tick attached, and collected blood and serum samples and examined them for TBPs, including *R. felis*, using molecular and serological assays. At the same time, when patients tested positive for TBPs, they implemented field studies near their residences to identify the components of the chain of TBP transmission to humans. One patient (74-year-old female) was found to be *R. felis* PCR positive; the patient presented with an adult female *Rh. sanguineus* s.l. tick attached and the approximate feeding time was 72 h. One week after the first presentation, the patient appeared with an enlarged and very painful occipital lymph node. The attached tick was negative for the presence of any of the TBPs tested, including *R. felis*, suggesting that the rickettsiae infection was not acquired from the *Rh. sanguineus* s.l. tick. During the field study implemented, there was no evidence of *R. felis* infection in the tested components; the blood samples collected from two dogs, and the tick samples removed from a cat, that the patient was in close contact with were found to be negative for *R. felis* [32].

##### Sweden

A single retrospective serological study from Sweden reported seroactivity against *R. felis* (detectable IgG titre 1:64) in one out of the 36 patients with facial nerve palsy that were included. The authors suggested that physicians in Sweden should consider a rickettsial infection upon the investigation and diagnosis of facial nerve palsy [35].

#### 2.2.2. Cats

##### Italy

The exposure and/or infection of cats to *R. felis* in central Italy was investigated during 2010–2016. The research team collected blood and buffy coat samples from 286 healthy cats from catteries and colonies, and used IFA to test for IgG against *R. felis* in serum samples and PCR to test for *R. felis* DNA in buffy coat samples. During visual inspection almost 57.34% and 8.04% of the cats had flea and tick infestation, respectively. In total, 8.04% (23/286) of the cats were seropositive for *R. felis* at a low titer (1:64). Co-exposure to *R. felis* and *R. conorii* or *Leishmania infantum* was detected in 5% and 3%, respectively. No *Rickettsia spp.* DNA was amplified using PCR. The authors found a significant association between seropositivity to *R. felis* and the origin of cats from catteries. Moreover, the cats ≥ 1 year of age and those infested with ectoparasites presented higher seropositivity rates against *R. felis*. However, the authors pointed out that considerable cross-reactivity exists between the various *Rickettsia* spp. of the SFG in IFA, and that in the absence of a PCR positive result, there is no solid evidence for the source of infection that stimulated the antibody response [36].

Later, Ebani et al. investigated the serological status against *R. felis* of 95 clinically healthy cats, infested by *C. felis*, in Tuscany, central Italy, from 2018 to 2021. The seropositivity to *R. felis* was 17.89% (17/95) when the cut-off used was 1/32, with titers ranging from 1/32–1/128. Overall, 58.94% of the cats had antibodies against at least one of the tested pathogens. In this study, four cats were found seropositive to both *R. felis* and *R. conorii* suggesting co-exposure or possible cross-reaction—although IFA is the gold standard method, cross-reactions cannot be ruled out [37].

##### Malta

Negative molecular results for *R. felis* detection were obtained in the study of Mifsud et al., which included blood samples from 23 clinically healthy adult cats from a shelter in the southern part of the island of Malta in 2017. However, as mentioned above in Section 2.1.1, the authors reported a high prevalence of *R. felis* infection in fleas collected from these cats [20].

##### Turkey

The 2021 study of Mustafa et al. provided the first evidence of *R. felis* in cats in Turkey. During 2017–2021, a high *R. felis* DNA prevalence of 26.3% was reported in 164 owned cats. It is worth mentioning that the cats included in this study were clinically ill; they were admitted to veterinary clinics with symptoms such as weight loss, fever, hematological abnormalities, and lymphadenopathy. Age and sex were not associated with *R. felis* infection status [38].

#### 2.2.3. Small Mammals

##### Germany

A study in Germany during 2012–2014 reported the occurrence of *R. felis* infection in small mammals. The investigation included 673 small mammals belonging to eight species (*A. agrarius*, *A. flavicollis*, *M. arvalis*, *M. agrestis*, *M. nivalis*, *M. glareolus*, *S. araneus*, and *T. europaea*). Overall, 25.3% (170/673) of the small mammals belonging to two species (the most represented in this study, *M. glareolus* and *A. flavicollis*) were positive for *Rickettsia* spp. DNA. Out of the 170 positive samples, 17 were sequenced; *R. felis* DNA was identified in one sample from *A. flavicollis* [27].

A later study, conducted from 2010 to 2014 in two different habitats in Germany, included 3939 tissue samples from small mammals. Molecular analysis showed that 8% (315/3939) of the samples were positive for *Rickettsia* spp. DNA. Out of the 77 samples that were sequenced, six were identical to *R. felis*; one in a European water vole (*A. amphibius*), four in yellow-necked mice (*A. flavicollis*) and one in a wood mouse (*A. sylvaticus*) [39].

##### Poland

A single study conducted in Poland in 2014 reported the detection of an *R. felis*-like organisms in a yellow-necked mice (*A. flavicollis*). The researchers collected samples from 193 wild rodents and the overall *Rickettsia* spp. prevalence recorded was 17.6% (34/193). However, only three sequences were successfully characterized at species level, two of them being identified as *R. helvetica* and one as an *R. felis*-like organism [40].

##### Slovakia

The first evidence of *R. felis* infection in *A. flavicollis* rodents in Slovakia came from a 2018 study by Heglasová et al. carried out during 2014–2016 in natural, suburban and urban habitats of the eastern part of the country. The researchers collected ear biopsies from 245 small mammals belonging to eight species: *A. agrarius*, *A. flavicollis*, *A. uralensis*, *M. glareolus*, *C. leucodon*, *M. minutus*, *M. arvalis* and *M. subterraneus*. The samples were molecularly examined and the overall *Rickettsia* spp. prevalence recorded was 11% (27/245). Out of the 27 positive samples, 11 were successfully sequenced; *R. felis* was detected in three *A. flavicollis* captured in a suburban habitat [41].

**Table 2:** The occurrence of *R. felis* in different hosts in Europe (2017–2022).

Countries	Study Period	Host	Prevalence in Host	Reference
Germany	2008	Human	2.7% (15/559) *	[34]
Germany	2010–2014	Wild mammals (*A. amphibious*, *A. flavicollis*, *A. sylvaticus*)	Not defined	[39]
Germany	2012–2014	Small mammals (*A. flavicollis*)	Not defined	[27]
Greece	2013	Human	3.5% (8/223) *	[16]
Italy	2010–2016	Cats	8.04% (23/286) *	[36]
Italy	2018–2021	Cats	17.89% (17/95) *	[37]
Malta	2017	Cats	0%	[20]
Poland	2014	Small mammals (*A. flavicollis*)	Not defined	[40]
Serbia	2019	Human	3% (1/30)	[31]
Serbia	2020	Human	Not defined (1/85)	[32]
Slovakia	2014–2015	Small mammals (*A. flavicollis*)	1.1% (3/27)	[41]
Sweden	2015	Human	Not defined *	[35]
Turkey	2017–2021	Cats	26.3% (44/167)	[38]

* Serological methods (IFA/MIF).

## 3. Discussion

In this review, we present the newest data on *R. felis* occurrence in vectors, animal and human hosts in European countries as reported during 2017–2022. European countries reported the detection of *R. felis* in several arthropod and host species: fleas, ticks and mites, and cats, small mammals and humans, respectively. Several studies provided the first evidence of *R. felis* detection in some countries, vectors or animal species, such as in *Ct. agyrtes* and *H. talpae* fleas and *H. microti* and *L. agilis* mites in Lithuania [18], fleas from cats in Malta [19], *Ct. b. boisseauorum* fleas in Spain [15], *Rh. turanicus* in Italy [28], cats in Turkey [38] and *A. flavicollis* in Slovakia [41]. In the studies conducted, *R. felis* positive fleas, ticks and mites were removed from different hosts: cats, dogs, hedgehogs, foxes, sheep, rodents, birds, small mammals *(A. agrarius*, *A. agrarius)* and humans, as well as from the environment (flagging) [17].

Among the flea species examined, *C. felis*, *C. canis*, *P. irritans*, *Ct. agyrtes*, *H. talpae*, *Ct. solutus*, *N. fasciatus*, *Ct. assimilis*, *A. erinacei* and *Ct. b. boisseauorum* were found to be infected with *R. felis*—with some of them being the first ever recordings [14,16,17,18,19,22,23,33,41]. Other flea species that have been found to be infected in previous studies include *C. orientis*, *Anomiopsyllus nudata*, *Ctenophthalmus* sp., *X. cheopis*, *X. brasilliensis*, *Tunga penetrans*, *Ceratophyllus gallinae*, *Spilospsyllus cuniculi* and *Echidnophaga gallinacean* [12,42,43].

Although numerous flea species have been found to be infected by *R. felis*, the cat flea is deemed as the primary vector of *R. felis*. Furthermore, the pathogen has been identified in the mid-gut, ovaries and salivary glands of *C. felis* suggesting that infection is disseminated within the arthropod [44]. Moreover, *R. felis* is transmitted transovarially and transstadially in cat fleas and vertical transmission of *R. felis* persists in *C. felis* for at least 12 generations without the aid of an *R. felis*-infected bloodmeal [45,46]. Moreover, this species in not host-specific and *R. felis*-infected individuals have been collected from numerous vertebrate species: cats, dogs, rodents, opossums, hedgehogs, horses, sheep, goats, gerbils, and monkeys [5,42,43,47]. The studies published the last five years showed that the prevalence of *R. felis* infection in fleas ranged considerably from approximately 1–96%, which is in agreement with previous studies suggesting a great variability among countries (for review see [6]).

Among the different tick species examined in the studies included, *R. felis* was detected most frequently in *I. ricinus*, but also in *Rh. turanicus* and *I. hexagonus.* Other tick species that were previously found to be infected in European countries, include *R. sanguineus* in Spain [48], *R. bursa* in Turkey [49], *Hae. sulcata* in Croatia [50] and *I. ricinus* in Germany [51].

A study in Slovakia showed that *I. ricinus* can harbor viable, infectious *R. felis* [52]. Moreover, a case of *R. felis* infection in an elderly patient parasitized by an *R. felis* positive adult *I. ricinus* female was reported in Serbia [31]. However, isolation of *R. felis* from clinical samples has not been achieved so far [53]. As shown recently, ticks exposed to *R. felis* maintained rickettsiae for one generation, but transmission was not stable [54]—the role of ticks in the epidemiology of *R. felis* needs further elucidation.

Similarly, the role of other *R. felis* positive arthropods in the epidemiology and transmission of this pathogen is unclear [43]. During the last five years in Europe, *R. felis* was also detected in *H. microti* and *L. agilis* mites while chigger (South Korea), mesostigmata mites (Taiwan) and the lice *Liposcelis bostrychophila* were previously found to be infected elsewhere [55,56,57].

Previous studies had shown that several host species, including cats, dogs, opossums, raccoons, rodents, and humans, were either seropositive or PCR positive for *R. felis* DNA. However, until now, a definitive host with appropriate clinical signs and bacteremia has not been identified [6,11,47]. The vertebrate hosts which were found to be *R. felis* infected or exposed during investigations in the last five years in Europe are cats (0–26.3%) [36,37,38], small mammals (1.1%) [41] and humans (2.7–3.5%) [16,31]. Free-roaming animals as well as the wild animals are of increased importance as they do not receive routine veterinary care as domestic cats and dogs do. Especially under certain circumstances that bring wildlife, free-roaming cats, and domestic animals in close proximity (e.g., when food is left outdoors), the potential for exchanging fleas and other ectoparasites increases [58].

*Rickettsia felis* is an emerging arthropod-borne pathogen which has been detected in a wide range of vectors and hosts worldwide. However, the role of the multiple arthropods that harbor the pathogen is still unclear; extensive field research, including of hosts and vectors close to the residences of *R. felis* human cases, would provide an insight into the components involved in the transmission chain. Clinicians should be aware of the epidemiology of the disease caused by *R. felis* and include it in the differential diagnosis of febrile disease with or without the presence of a rash. Additionally, clinicians should be well-informed about the possible arthropod species that could harbor *R. felis* and include information on exposure to these vectors during data collection of the clinical case history. As for pets, veterinarians should keep training pet owners on the need for effective year-round arthropod control, especially for fleas, on their pets and in the environment.

## Data Availability

Not applicable.
